# Acute exacerbations of copd (AECOPD) in the emergency room (ER): prognostic value of proadrenomedulline (PROADM)

**DOI:** 10.1186/2197-425X-3-S1-A384

**Published:** 2015-10-01

**Authors:** M Dres, P Hausfater, J-M Treluyer, F Foissac, A-L Philippon, L-M Joly, M Sebbane, P Plaisance, N Roche, Scientific Committee and Investigators of the UTAPE Study

**Affiliations:** Assistance Publique Hôpitaux de Paris, Respiratory and Critical Care Department, Hopital Pitie Salpetriere, Paris, France; Assistance Publique Hôpitaux de Paris, Emergency Department, Hopital Pitie Salpetriere, Paris, France; Unité de Recherche Clinique, Hôpital Tarnier, Assistance Publique Hôpitaux de Paris, Paris, France; Emergency Department, Centre Hospitalier Universitaire Charles Nicolle, Rouen, France; Emergency Department, Centre Hospitalier Régional Universitaire de Montpellier, Montpellier, France; Assistance Publique Hôpitaux de Paris, Emergency Department, Hopital Lariboisiere, Paris, France; Assitance Publique Hôpitaux de Paris, Respiratory Care, Hopital Cochin, Paris, France

## INTRODUCTION

AECOPD represent an frequent cause of emergency room visits and hospital admissions. Prognostic tools are needed to guide treatment and orientation decisions.

## Objectives

The purpose of the study was to assess whether proADM level on admission added to the clinical assessment in the ER for predicting AECOPD outcome.

## Methods

This French prospective multicenter observational study was conducted in 22 hospitals from March 2013 to September 2014. All patients admitted to the ER with AECOPD as primary diagnosis were considered for inclusion. A previously published clinical prognostic score was computed based on the presence of clinical severity signs at entry, baseline dyspnea grade and age [[1]]. This score allows classification of patients in 3 risk categories (high, intermediate and low). Venous blood sample was obtained for duplicate determination of proADM level. The primary endpoint was a composite criteria comprising 30-day mortality, secondary transfer to an intensive care unit and AECOPD recurrence. The primary analysis was the assessment of the predictive value of proADM for the primary endpoint using a multivariate logistic regression model adjusted for the clinical risk category.

## Results

Three hundred seventy two patients (69.7 ± 11.5 years) were consecutively enrolled. Overall, 277 (75%) met the primary composited end point. More specifically, the primary composite end point was encountered respectively in 7 (16%), 24 (21%) and 35 (29%) patients in the low, intermediate and high-risk categories, respectively. The mean ( ± sd) admission proADM level was 0.75 ( ± 0.25), 0.98 ( ± 0.61) and 1.2 ( ± 0.73) nmol/L in the low, intermediate and high-risk categories respectively (p < 0.0001). ProADM level at admission was an independent predictor of outcome after adjustment for the clinical risk category, OR [IC95] 1.7 (1.1 - 2.7), p < 0.05.

## Conclusions

These preliminary data show that proADM predicts poor outcome even after adjustment for the clinical score. This suggests that a new prognostic rule combining clinical features and proADM level would be useful.

## Grant Acknowledgment

This study was funded by BRAHMS - ThermoFischer
